# Encephaloid Cancer in a Child Two Years and Seven Months Old

**Published:** 1877-08

**Authors:** T. Davis Fitch


### ENCEPHALOID CANCER IN A CHILD TWO YEARS AND SEVEN MONTHS OLD.

By Prof. T. DAVIS FITCH.

February 18th, 1877, I was called to see Theodore L. B., aged two years and seven months. He was a robust child, large of his age—dark complexion, and had always enjoyed extraordinary good health from his birth, with the exception of slight bowel derangement during dentition, and a green-stick fracture of the left ulna in July, 1876. The child was delivered under my care, and had always been treated by me in these slight derangements, so that I had been familiar with him from his birth, and know that he was an unusually robust child.

On examination at the date above mentioned, I found the child irritable in temper, fretful, temperature slightly elevated, pulse 120, bowels constipated, urine normal. His face was pallid and his eyes sunken aud glassy, with a dark ring under them; his countenance resembling that of a child in collapse, from cholera-infantum.

February 19.—Found the child in much the same condition as on the previous visit. The mother called my attention to the fact that the child had been a little lame in the right leg, and this was verified by allowing him to walk across the room. I then pressed the head of the femur with some force into the acetabulum accompanied with rotation, and it seemed to give him pain, though there was apparently no inversion or eversion of the foot; it led me to suspect morbus coxarius. Bowels had moved and some slight relief seemed to be afforded.

February 22d.—The condition very little changed. Noticed an unusual prominence of the right cheek, which seemed to be hard and insensible. No pain manifested on pressure, no abnormal heat or redness. Was informed that the child fell against the corner of a chest about two weeks previous to this attack, but no mark of the bruise had resulted; no pain complained of. The child had unusually high cheek bones, and this was thought due to irregularity of the features.

February 25th.—The child seemed worse, and the swelling of the cheek had increased. I then examined the mouth and found a swelling in the gum opposite the eye tooth, which I then thought was an abscess, from its doughy feel or semi-fluctuating character. On puncturing, it discharged only blood. I was then led to fear malignant disease, and expressed my convictions of this fact to the parents. *No history of cancer in the family.*

February 26th.—Called and found the child in much the same condition but evidently failing; advised counsel.

February 27th.—Dr. R. G. Bogue was called, the diagnosis was confirmed, and an unfavorable prognosis given.

The buccal tumor continued to grow until about March 12th, when it protruded an inch beyond the lips, and in a few days sloughed off. The cheek became more prominent, glassy, and the skin so thin and transparent that the enlarged subcutaneous vessels could be very distinctly seen. About this time Prof. Danforth was called in, who at once recognized the disease and corroborated the diagnosis and prognosis. The child became quite prominent in the epigastric region, and on palpation a hard tumor was detected reaching nearly to the ensiform cartilage; this swelling increased rapidly till his death on the 27th March.

Autopsy 24 hours after death. Present Profs. Danforth, Chas. W. Earle and Sarah Hackett Stevenson. The child was greatly emaciated. The main object of the post mortem was to ascertain the origin and character of the tumor in the abdominal cavity. No examination was made of the facial tumor. The lungs, pleura liver, heart, stomach and intestines were found in a healthy condition.

The tumor was found to be a cancerous degeneration of the left kidney; not even a trace of the normal kidney remaining. The tumor was composed of cysts of various sizes and containing fluid of various kinds; large clots of blood were found in some of them. A tumor of similar character was found in the right groin about the iliac vessels, immediately above Poupart's ligament. This tumor was somewhat larger than a hen's egg, and was undoubtedly the cause of the lameness complained of in the first week of the sickness.

The larger tumor was about the size of a man's two fists, and would weigh about two pounds.

These specimens were place in the hands of Dr. Danforth for examination and presentation. He will give you the *microscopy*.

*Note.*—The histological elements found in the specimens placed in my hands by Prof. Fitch were such as clearly indicated encephaloid cancer of the most virulent or rapidly growing form. Large atypical cells were abundant, but the connective tissue element or

"stroma" was almost wholly wanting. The cells presented no great variety as to form, because the density of the cancerous mass being much the same in all its parts, they encountered the same resistance to growth on all sides; hence it was possible for each cell to develop symmetrically. In scirrhus and the denser and more slowly growing forms of encephaloid, the developing cells encounter fibres of connective tissue between which they must push their wedge shaped processes; therefore, we find the "caudate" or "polygonal" cells, which were so long regarded as indicative of cancer. But the cells in the case under consideration presented a very marked variety as regards size and other features. The number of nuclei varied from one to four; and some of them, might well have been taken for the "giant cells" of Virchow. I found the usual luxuriant small cell infiltration, derived from the leucocytes and their descendants; also the *debris* of degenerating structures which were invaded by the cancerous growth, and numerous fat globules. It seems to me that Prof. Fitch's case is one of profound interest pathologically. A child only two years old, of fine *phisique*, enjoying "extraordinary good health," reared under favorable circumstances as regards care, cleanliness, diet, air and all other conditions which conduce to infantile health; entirely innocent of any hereditary tendency to cancerous disease, so far as could be ascertained, and quite too young to have acquired any such disease by means of personal vices, is suddenly attacked by cancer in its most virulent and ferocious form. Moreover, it is a case so clearly typical, that no question can be raised as to its nature; every one of us who saw the child either ante- or post-mortem, at once pronounced the disease cancer, and the microscope added its confirmatory evidence. The question of all-absorbing interest to the pathologist is, whence and how came this invasion of cancer in a manner apparently so causeless and inexplicable? Such cases teach us that pathology is yet in its infancy.
I. N. D.

Reported July 30th, 1877.

